# m^6^A RNA Methylation Regulators Contribute to Malignant Progression and Have Clinical Prognostic Impact in Gastric Cancer

**DOI:** 10.3389/fonc.2019.01038

**Published:** 2019-10-18

**Authors:** Yunshu Su, Jinqi Huang, Jichang Hu

**Affiliations:** ^1^Department of Thoracic Surgery, Renmin Hospital of Wuhan University, Wuhan, China; ^2^Division of Cardiothoracic Surgery, Central Hospital of EnShi Tujia and Miao Autonomous Prefecture, EnShi Clinical College of Wuhan University, EnShi, China; ^3^Department of Pathology, Renmin Hospital of Wuhan University, Wuhan, China

**Keywords:** gastric cancer, m^6^A, TCGA, epigenetic modification, FTO

## Abstract

N6-methyladenosine (m^6^A) is the most common form of mRNA modification, and is dynamically regulated by the m^6^A RNA methylation regulators. However, little is known about m^6^A in gastric cancer. The aim of this work is to investigate the effects of m^6^A RNA methylation regulators in gastric cancer. Here, we found that most of the 13 main m^6^A RNA methylation regulators are higher expressed in 375 patients with gastric cancer. We identified two subgroups of gastric cancer (cluster1 and 2) by applying consensus clustering to m^6^A RNA methylation regulators. Compared with the cluster1 subgroup, the cluster2 subgroup correlates with a poorer prognosis, and most of the 13 main m^6^A RNA methylation regulators are higher expressed in cluster2. Moreover, the cancer-specific pathways are also significantly enriched in the cluster2 subgroup. This finding indicates that m^6^A RNA methylation regulators are closely associated with gastric cancer. Based on this finding, we derived a risk signature, using 3 m^6^A RNA methylation regulators (FTO, RBM15, ALKBH5), that is not only an independent prognostic marker but can also predict the clinicopathological features of gastric cancer. Moreover, FTO is higher expressed in high risk scores subtype in gastric cancer. Thus, this first finding provide us clues to understand epigenetic modification of RNA in gastric cancer.

## Introduction

N6-methyladenosine (m^6^A) is a methylation modification that can occur on RNA adenine (A) (1). Of the 171 known RNA post-transcriptional modifications (2), m^6^A is one of the most abundant modifications in most eukaryotic mRNA and lncRNA, accounting for 0.1–0.4% of adenylate and 50% of total ribonucleotides in mammalian RNA (3, 4). In addition to the extensive m^6^A modification in plants and vertebrates, this modification has also been found in single-celled organisms such as bacteria and yeast (5, 6). m^6^A modification mainly occurred in the common sequence of RRACH (R = G or A, H = A, C, or U) (7, 8). Through high throughput sequencing, it was found that m^6^A was not randomly distributed. Instead, it was aggregated in the stop codon, 3′ untranslated region (3′UTR), and internal exons (9–11), and more were found in the precursor mRNA (12). More and more studies have shown that m^6^A modification plays an important role in the occurrence and development of human complex diseases, especially in the occurrence and development of cancer (13–15).

Through the study of m^6^A related proteins, it is found that m^6^A methylation is a dynamic reversible process (16), which is composed of methyltransferase complex (writers), demethylase (erasers), and function manager (readers) (17). Writers is a process of “writing” methylated modifications into RNA, that is, mediating the process of methylated modification of RNA, including METTL3, METTL14, KIAA1429, WTAP, RBM15, and ZC3H13 (18).Erasers can “erase” the RNA methylation modification signal, that is, mediating the demethylation process of RNA, including FTO and ALKBH5 (19, 20). Readers is responsible for “reading” RNA methylated information and participating in the translation and degradation of downstream RNA, including YTHDC1, YTHDC2, YTHDF1, YTHDF2, and HNRNPC (21). m^6^A, under the influence of the “writer,” adds methyl groups to RNA, and recognizes those m^6^A-modified RNAs through different “readers” to produce different functions, including RNA processing, nuclear export, translation, and decay. Finally, relying on the role of “Erasers,” the process of m^6^A modification becomes dynamic and reversible, thereby functioning to regulate the expression of various genes (14).

Due to RNA regulation is closely related to human diseases, as one of the most abundant internal modifications in mammalian cells, m^6^A methylation modification has been confirmed with various diseases such as obesity (22), diabetes (23), infertility (24), tumor (25), and neuronal diseases (26). However, little is known about m^6^A in gastric cancer. In this study, we systematically analyzed the expression of 13 widely reported m^6^A RNA regulators in 375 gastric cancer with RNA sequencing data from The Cancer Genome Atlas (TCGA) datasets, as well as the association between clinicopathological characteristics.

## Materials and Methods

### Data Acquisition

The RNA-seq transcriptome data and corresponding clinical information of STAD cohort were downloaded from TCGA (https://cancergenome.nih.gov/) data portal (level 3). All mRNASeq gene expression data are downloaded through the R package “TCGA-Assembler.”

### Selection of m^6^A RNA Methylation Regulators

There are 13 genes in the m^6^A RNA methylation regulator. We extracted the expression matrix of these 13 genes and the clinical information of the sample. The extracted information is used for subsequent bioinformatics analysis.

### Bioinformatic Analysis

To investigate the function of m^6^A RNA methylation regulators in gastric cancer, we used Limma package to analyze the expression of 13 genes in 375 tumor patients and 32 normal gastric tissue. The upper tree diagram represents clustering results for different samples from different experimental groups, and the left tree shows cluster analysis results for different genes from different samples. Next, we used a vioplot to visualize the expression of 13 genes in 375 tumor patients and 32 normal gastric tissue. The white point represents the median Q2 (half of the data is greater than the median, above it, and the other half is less than the median, below it). The black rectangle is the range from the lower quartile to the upper quartile. The upper edge of the rectangle is the upper quartile Q3, which means that one quarter of the data is larger than the upper quartile, and the lower edge is the lower quad. The quantile Q1 represents that one quarter of the data is less than the lower quartile. The length of the interquartile range IQR (the upper quartile and the lower quadrant) represents the dispersion and symmetry of the non-abnormal data. The length is scattered and the short is concentrated. The black line running through the violin map represents the minimum non-abnormal value min. To the interval of the maximum non-outlier max, the lower and upper limits represent the upper and lower limits, respectively, and the range is beyond the abnormal data; the outer shape of the black rectangle is the kernel density estimation, the length of the vertical axis of the graph represents the degree of data dispersion, and the length of the horizontal axis represents the Data distribution of an ordinate position.

Next, we removed 32 normal tissue samples and grouped 375 cancer tissues using the ConsensusClusterPlus package, using PCA to verify the results of the grouping. GO and KEGG analysis of genes with different expression of cluster2 relative to cluster1 using GOplot package. Finally, we use the survival package to analyze the survival of the cluster, and we performed univariate Cox regression analyses of their expression in the TCGA dataset.

### Statistical Analyses

One-way ANOVA was used to compare the expression level of 13 genes in 375 tumor patients and 32 normal gastric tissue in TCGA dataset, and *t*-tests were used to compare the expression levels in gastric cancer for age, gender, stage, T status, M status, and N status. Overall survival (OS) is defined as the interval from the date of diagnosis to the date of death. Before constructing the scoring model, we first obtain the optimal cut-off value of each risk score in the training group through the “survminer” package in the software, and divide the cells into high and low groups according to the best cutoff value, and was represented by 1.0. Cox regression analysis was used to evaluate the association between risk score and OS, in which age and sex were used as covariates. The missing data is processed by list deletion, and if any single value is missing, the entire sample is excluded from the analysis. Using R version 3.5 for all statistical analysis, *P* < 0.05 was statistically significant.

## Results

### The Landscape of m^6^A RNA Methylation Regulators in Gastric Cancer

**Figure 1:**
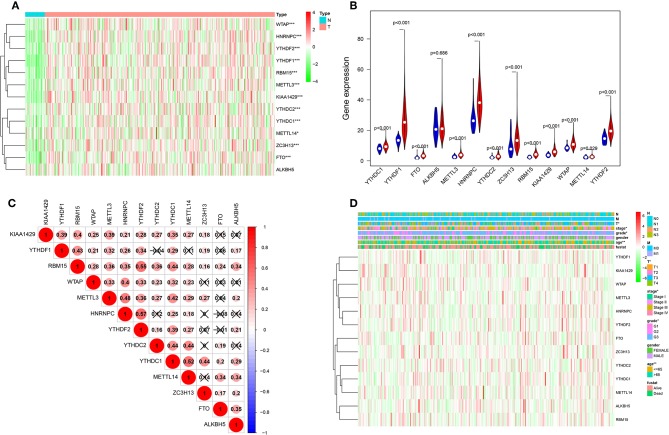
The landscape of m^6^A RNA methylation regulators in gastric cancer. **(A)** The expression levels of 13 m^6^A RNA methylation regulators in gastric cancer. The higher or lower the expression, the darker the color (red is up-regulated and green is down-regulated). The upper tree diagram represents clustering results for different samples from different experimental groups, and the left tree shows cluster analysis results for different genes from different samples. **(B)** Vioplot visualizing the differentially m^6^A RNA methylation regulators in gastric cancer (assume blue is normal and red is gastric cancer). **(C)** Spearman correlation analysis of the 13 m^6^A modification regulators in gastric cancer. **(D)** Expression of m^6^A modification regulators in gastric cancer with different clinicopathological features. ^*^P < 0.05, ^**^P < 0.01, and ^***^P < 0.001.

Considering the important biological functions of each m^6^A RNA methylation regulator in tumorigenesis and development. We first compare the expression level of 13 m^6^A RNA methylation regulators in 375 gastric cancer tissues and 32 normal gastric tissue in TCGA dataset. Compared with normal gastric tissue, gastric cancer patients generally contain a higher proportion of METTL3, METTL14, WTAP, KIAA1429, RBM15, ZC3H13, YTHDC1, YTHDC2, YTHDF1, YTHDF2, HNRNPC, and FTO (Figures 1A,B). We speculate that the change of m^6^A RNA methylation regulators ratio may be an intrinsic feature that can characterize individual differences, Figure 1C showed the proportion of different m^6^A RNA methylation regulators is weakly to moderately correlated. The relationship between the 13 m^6^A RNA methylation regulators is positively correlated, and the YTHDF2 gene and the RBM15 gene are most relevant. When the YTHDF2 gene is up-regulated, the RBM15 gene is most likely to be up-regulated (Figure 1C). We also systematically investigated the relationships between each individual m^6^A RNA methylation regulator and the pathological features of gastric cancer, including age, gender, grades, stage status, T status, M status, and N status, and found there is relationship between m^6^A RNA methylation regulator and pathological features of gastric cancer (Figure 1D).

### Consensus Clustering of m^6^A RNA Methylation Regulators Identified Two Clusters of Gastric Cancer

**Figure 2:**
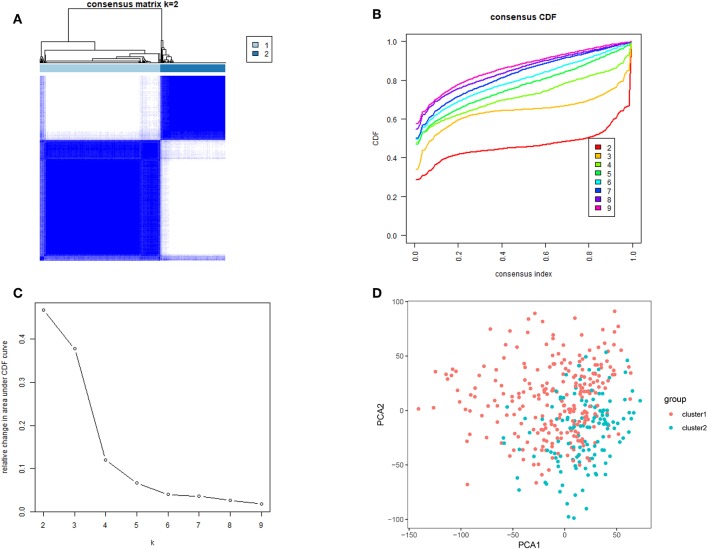
Identification of consensus clusters by m^6^A RNA methylation regulators. **(A)** Consensus clustering matrix for *k* = 2; **(B)** consensus clustering cumulative distribution function (CDF) for *k* = 2–9; **(C)** relative change in area under CDF curve for *k* = 2–9; **(D)** principal component analysis of the total RNA expression profile in the TCGA dataset. Gastric cancer in the cluster1 subgroup are marked with red, and the cluster2 subgroup are marked with blue.

Next, we removed 32 normal gastric tissue samples and grouped 375 cancer tissues using the ConsensusClusterPlus package. Based on the expression similarity of m^6^A RNA methylation regulators, *k* = 3 seemed has smaller CDF value in the TCGA datasets (Figures 2B,C), however, after being divided into three groups, the correlation between the groups is high, and there is a small number of samples. Therefore, we are divided into two groups (Figure 2A). In order to judge whether our classification is correct, we will analyze the two subclasses by PCA, and the results show cluster 1 can gathered together and cluster 2 can also be gathered together (Figure 2D). These results indicate that the results of our classification by m^6^A RNA methylation regulators are correct.

### Categories Identified by Consensus Clustering Are Closely Correlated to Clinical Outcomes and Clinicopathological Features

**Figure 3:**
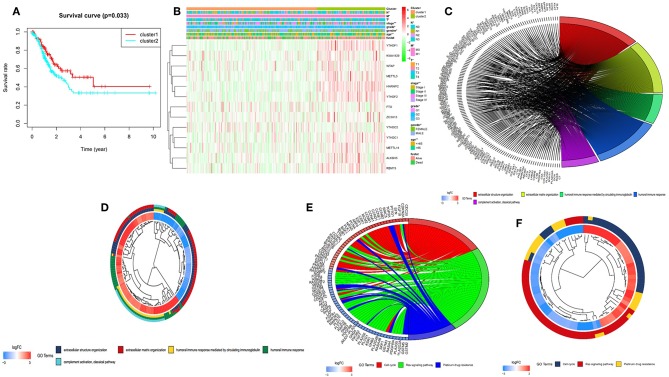
Differential clinicopathological features and overall survival of gastric cancer in the cluster 1/2 subgroups. **(A)** Kaplan–Meier overall survival (OS) curves for 375 TCGA gastric cancer patients. Gastric cancer patients in the cluster1 subgroup are marked with red, and the cluster2 subgroup are marked with blue. **(B)** Heatmap and clinicopathologic features of the two clusters (cluster1/2) defined by the m^6^A RNA methylation regulators consensus expression. The higher or lower the expression, the darker the color (red is up-regulated and green is down-regulated). The upper tree diagram represents clustering results for different samples from different experimental groups, and the left tree shows cluster analysis results for different genes from different samples. **(C–F)** Functional annotation of the genes with higher expression in the clusters 2 subgroup using GO terms of biological processes **(C,D)** and KEGG pathway **(E,F)**. ^*^P < 0.05, ^**^P < 0.01.

To better understand the clustering result and clinical outcomes and clinicopathological features, we analyzed the clustering result and OS curves for 375 gastric cancer patients. We found the cluster 2 subgroup has a significantly shorter OS than the cluster 1 subgroup (Figure 3A). Moreover, we found that most of m^6^A RNA methylation regulators have high expression in cluster 2 subgroup. Compare with the cluster 1 subgroup, the cluster 2 subgroup is significantly correlated with older age at diagnosis at diagnosis, higher grade, higher stage, higher T status, higher M status, and higher N status (Figure 3B). According to the evidence, the clustering result was closely correlated to the malignancy of the gastric cancer. To better understand the clustering result and their function, we analyzed GO and KEGG analysis of genes with different expression of cluster2 relative to cluster1 using GOplot package. Go results indicated that upregulated genes are enriched in malignancy-related biological processes, including extracellular structure organization, extracellular matrix organization, humoral immune response, humoral immune response mediated by circulating immunoglobulin, and complement activation, classical pathway (Figures 3C,D). KEGG results indicated that upregulated genes are enriched in cell cycle, ras signaling pathway and platinum drug resistance (Figures 3E,F).

### Prognostic Value of Risk Signature and m^6^A RNA Methylation Regulators

**Figure 4:**
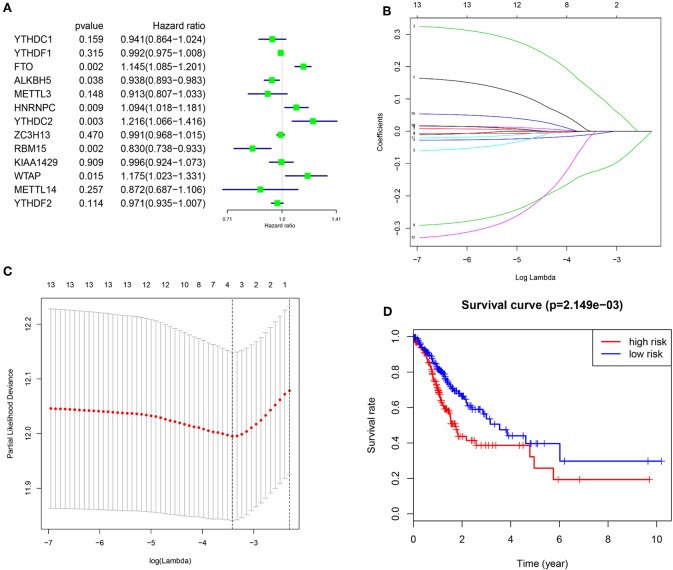
Risk signature with three m^6^A RNA methylation regulators. **(A)** The process of building the signature containing 13 m^6^A RNA methylation regulators. The hazard ratios (HR), 95% confidence intervals (CI) calculated by univariate Cox regression. **(B,C)** The coefficients calculated by multivariate Cox regression using LASSO are shown. **(D)** Kaplan–Meier overall survival (OS) curves for patients in the TCGA datasets assigned to high and low risk groups based on the risk score.

To better understand the prognostic role of m^6^A RNA methylation regulators in gastric cancer, we performed a univariate Cox regression analysis on the expression levels in the TCGA dataset. The results indicated that high expression of FTO (HR = 1.15, 95% CI = 1.02–1.29), HNRNPC (HR = 1.09, 95% CI = 1.02–1.18), YTHDC2 (HR = 1.22, 95% CI = 1.07–1.42), and WTAP (HR = 1.18, 95% CI = 1.02–1.33) have a worse survival in patients with gastric cancer. In contrast, high expression of ALKBH5 (HR = 0.94, 95% CI = 0.89–0.98) and RBM15 (HR = 0.83, 95% CI = 0.74–0.93), have a better survival in patients with gastric cancer (Figure 4A).

In order to predict the clinical outcomes of gastric cancer with m^6^A RNA methylation regulators, we applied the least absolute shrinkage and selection operator (LASSO) Cox regression algorithm to the 13 genes in the TCGA dataset. Three genes (FTO, ALKBH5, and RBM15) were selected to build the risk signature based on the minimum criteria, and the coefficients obtained from the LASSO algorithm were used to calculate the risk score for TCGA dataset (Figures 4B,C). To investigate the prognostic role of the three-gene risk signature, we separated the gastric cancer patients in TCGA dataset into low and high-risk groups based on the median risk score, the results indicated that high-risk group have a worse survival in patients with gastric cancer (Figure 4D).

### Prognostic Risk Scores Showed Strong Associations With Clinicopathological Features in Gastric Cancer

**Figure 5:**
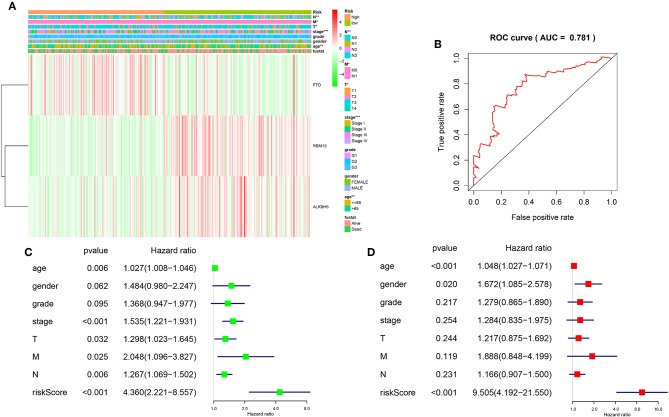
Relationship between the risk score, clinicopathological features, and clusters subgroups. **(A)** The heatmap shows the expression levels of the three m^6^A RNA methylation regulators in low and high risk gastric cancer patients. The distribution of clinicopathological features was compared between the low- and high-risk groups. **(B)** ROC curves showed the predictive efficiency of the risk signature. **(C)** Univariate Cox regression analyses of the association between clinicopathological factors (including the risk score) and overall survival of patients in the TCGA datasets. **(D)** Multivariate Cox regression analyses of the association between clinicopathological factors (including the risk score) and overall survival of patients in the TCGA datasets. ^*^P < 0.05, ^**^P < 0.01, and ^***^P < 0.001.

In order to better understand the clinical outcomes of gastric cancer with high-risk groups, we systematically investigated the relationships between the three selected m^6^A RNA methylation regulators in high risk group and low risk group patients in the TCGA dataset and the pathological features of gastric cancer, including age, stage status, T status, M status, and N status, and found there is relationship between three selected m^6^A RNA methylation regulators in high risk group and low risk group patients and pathological features of gastric cancer (Figure 5A). Moreover, compare with low risk group patients, gastric cancer patients generally contain a higher proportion of FTO, lower proportion of ALKBH5 and RBM15 in the high risk group (Figure 5A).

To better understand the relationships between risk scores and gastric cancer patients, firstly, we do a ROC curve to predict risk scores and 3-year survival rates for gastric cancer patients, the results indicated that the risk score can predict 3-year survival rates for gastric cancer patients (AUC = 0.781) (Figure 5B). Next, we performed univariate and multivariate Cox regression analyses for the TCGA dataset to determine whether the risk signature is an independent prognostic indicator. Both the univariate and multivariate Cox regression analyses results indicated that the risk score, age, stage status, T status, M status, and N status were all correlated with the OS. As the risk score, age, stage status, T status, M status, and N status increases, the risk increases (Figures 5C,D). According to the evidence, prognostic risk scores showed strong associations with clinicopathological features in gastric cancer, and FTO was correlated with the malignancy of gastric cancer.

### FTO Showed High Expression in Human Tissues

**Figure 6:**
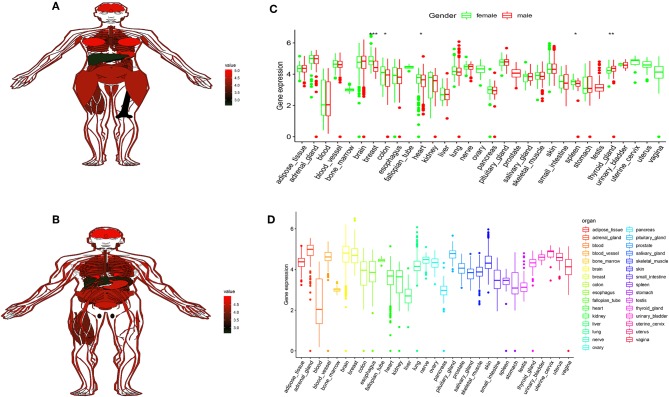
FTO showed high expression in human tissues. **(A,B)** The map shows the expression levels of the FTO in the 31 solid organ tissues in female and male. **(C)** Histogram visualizing the differentially FTO in the 31 solid organ tissues in female and male. **(D)** Histogram visualizing the differentially FTO in the 31 solid organ tissues. ^*^P < 0.05, ^**^P < 0.01, and ^***^P < 0.001.

To better understand FTO in human tissues, we used GTEx (Genotype-tissue expression) dataset to know FTO expression differs among different tissues and individuals. The GTEx database contains more than 7,000 autopsy samples from 449 pre-healthy human donors, covering 44 organizations (42 different tissue types), including 31 solid organ tissues, 10 brain regions, whole blood, and 2 from donor blood and skin cell lines. The results indicated higher of FTO expression was found in the 31 solid organ tissues (Figure 6D) and in female and male (Figures 6A,B). In most female and male tissues, there is no difference in the expression of FTO, and there were significantly differences in breast, colon, spleen, and thyroid (Figure 6C).

## Discussion

Gastric cancer is the fifth largest malignant tumor in the world, which is a serious threat to human health and life safety (27). Surgery is the first choice for the treatment of gastric cancer, combined with adjuvant chemotherapy, radiotherapy, targeted drugs, and immunotherapy (28). Although, the global incidence of gastric cancer has declined significantly over the past few decades, the 5-year survival rate of gastric cancer is usually <30%, and there are still many key issues that remain unresolved (29). The occurrence and development of gastric cancer is very complicated. It is a multi-factor, multi-step complex process involving external environmental factors, diet, living habits, and also involves tissue cell differentiation, genetic changes, cell cycle changes, metabolism, gene expression, molecular interaction, signal transduction pathway changes, it is also related to host immune status, homeostasis and other factors (30). Although targeted therapy can prolong the survival of patients, tumor drug resistance and economic burden are considerable problems in clinical practice (31). Therefore, exploring the molecular mechanisms of gastric cancer pathogenesis and new therapeutic targets remains a challenging issue.

m^6^A, as a member of RNA epigenetic modification families, is not “good or bad” based on the current understanding of m^6^A and tumor. It can promote or inhibit tumor cells mainly by regulating the mRNA expression of related oncogenes or tumor suppressor genes. The m^6^A methylation site appeared in the nuclear RNA under the action of Writers. The m^6^A methylation site of RNA in the nucleus can also be erased under the action of erasers. Subsequently, in the further processing of the nuclear RNA, the readers (reading protein) in the nucleus will bind to the m^6^A methylated site; when the mature RNA comes out of the nucleus, there will still be some readers outside the nucleus will bind to its m^6^A site. It is worth noting that different Reader binding to m^6^A will produce different biological effects (14). The methylation level of m^6^A is closely related to the expression level of intracellular writing and erasing genes, while the protein molecules that read gene expression are combined with the m^6^A methylation site to perform a series of biological functions (32). Therefore, in tumors, both m^6^A-related genes and protein expression levels may become potential markers for tumor molecular diagnosis, and will also provide new targets for the development of clinical molecular targeted therapeutic drugs.

This study attempted to the effects of m^6^A RNA methylation regulators in gastric cancer, and found m^6^A RNA methylation regulators was closely associated with pathological features of gastric cancer. We identified two subgroups of gastric cancer by applying consensus clustering to m^6^A RNA methylation regulators, and the cluster 2 subgroup correlates with a poorer prognosis. In addition, we derived a risk signature by using 3 m^6^A RNA methylation regulators. The risk score is not only an independent prognostic marker but can also predict the clinicopathological features of gastric cancer. Moreover, FTO is higher expressed in high risk scores subtype in gastric cancer. According to the evidence, FTO was correlated with the malignancy of gastric cancer.

FTO was originally reported as a demethylase for N3-methylthymidine in single-stranded DNA and for N3-methyluridine in single-stranded RNA *in vitro*. Depletion of FTO induces significant increase in total m6A levels of polyadenylated RNA. As FTO oxidizes m6A to A, it generates N6-hydroxymethyladenosine (hm6A) as an intermediate product, and N6-formyladenosine (f6A) as a further oxidized product. The potential function of these oxidized labile intermediates needs further exploration (17). Li et al. found that in acute myeloid leukemia (AML), high expression of FTO can reduce the level of m^6^A methylation in the mRNA of ASB2 and RARA genes, which leads to the occurrence and development of AML, and it was found that high expression of FTO could inhibit the differentiation of AML cells into normal blood cells mediated by all-trans-retinoic acid (33). This makes the FTO demethylation gene an oncogene for AML. Zhou et al. found a significant increase in the expression of FTO in tumor tissues of patients with cervical squamous cell carcinoma (CSCC), and found that these patients developed tolerance to radiotherapy and chemotherapy. This may be due to the fact that FTO reduces the m^6^A methylation level of certain genes, thereby activating the β-catenin pathway and affecting the expression of ERCC1 genes. In addition, it was also found that both FTO and β-catenin expression in CSCC patients showed a worse prognosis than patients who were elevated alone (*P* = 0.041). Thus, the expression of FTO and β-catenin has certain value in evaluating the clinical prognosis of CSCC (34).

Tumor stem cells are a kind of pluripotent tumor cells, which are highly malignant and have the ability of self-renewal to mutate more quickly to produce drug resistance or adapt to changes in the microenvironment. It has been found that a certain number of m^6^A methylation and tumor studies are related to tumor stem cells (35–37). Cui et al. found that the use of FTO inhibitors can significantly inhibit the growth of glioblastoma stem cells (GSC) and reduce the frequency of transformation of GSC cells into tumor stem cells. Moreover, the use of MA2 in glioblastoma can effectively inhibit FTO expression and inhibit tumor progression. This also provides guidance for people looking for new targeted drugs (38). The above studies emphasize the importance of FTO and provide evidence for exploring the pathogenesis of some tumors and seeking new potential therapeutic targets by revealing the previously unconfirmed mechanism of tumor gene regulation. It also provides a new idea for the mechanism of tumor epigenetic modification and tumor gene targeting therapy.

However, to date, many FTO inhibitors (rheumine, IOX3, and meclofenamic acid) have been reported, most of which are not specific. Meclofenamic acid can stably bind to FTO, but the effect on ALKBH5 is still in the research stage (39). IOX3 is a HIF proline hydroxylase inhibitor, which can bind to the active site of FTO and reduce the expression level of FTO, but the inhibitor failed to alter the level of intracellular m^6^A (40). So far, the role and specific mechanism of m6A demethylase inhibitors found in *in vitro* and *in vivo* studies are not fully understood and lack specificity. Therefore, researchers are expecting more inhibitors against m^6^A-related factors, especially more specific inhibitors, to bring new dawn to guide tumor gene targeting therapy.

In conclusion, our results systematically demonstrate the expression, potential function, and prognostic value of m^6^A RNA methylation regulators in gastric cancer. The expression of m^6^A RNA methylation regulator is highly correlated with the malignant clinicopathological features of gastric cancer. Our study provides important evidence for future detection of the role of m^6^A methylation in gastric cancer.

## Data Availability Statement

The data used to support the findings of this study are from the cancer genome map of the public database (the cancer genome atlas, TCGA, https://cancergenome.nih.gov/).

## Author Contributions

JicH designed the research, analyzed the data and wrote the paper. YS performed the data analysis and interpreted the data. JinH help to revised manuscript the article. All authors read and approved the final manuscript.

### Conflict of Interest

The authors declare that the research was conducted in the absence of any commercial or financial relationships that could be construed as a potential conflict of interest.
